# The cost-effectiveness of osteochondral allograft transplantation in the knee

**DOI:** 10.1007/s00167-019-05392-8

**Published:** 2019-02-05

**Authors:** Hema Mistry, Andrew Metcalfe, Nick Smith, Emma Loveman, Jill Colquitt, Pamela Royle, Norman Waugh

**Affiliations:** 1 Division of Health Sciences, Warwick Medical School, University of Warwick, Gibbet Hill Road, Coventry, CV4 7AL UK; 2 Warwick Clinical Trials Unit, Warwick Medical School, University of Warwick, Coventry, CV4 7AL UK; 3 University Hospitals Coventry and Warwickshire, Coventry, CV2 2DX UK; 4 Effective Evidence LLP, 26 The Curve, Waterlooville, Hampshire PO8 9SE UK

**Keywords:** Allografts, Osteochondral, Systematic review, Cost-effectiveness

## Abstract

**Purpose:**

Osteochondral allografts (OCA) consist of a layer of hyaline cartilage and a layer of underlying bone. They are used to repair combined defects of articular cartilage and bone. Such defects often occur in people far too young to have knee arthroplasty, for whom the main alternative to OCA is conservative symptomatic care, which will not prevent development of osteoarthritis. The aim of this report was to assess the cost-effectiveness of osteochondral allograft transplantation in the knee.

**Methods:**

Systematic review of evidence on clinical effectiveness and economic modelling.

**Results:**

The evidence on osteochondral allograft transplantation comes from observational studies, but often based on good quality prospective registries of all patients having such surgery. Without controlled trials, it was necessary to use historical cohorts to assess the effect of osteochondral grafts. There is good evidence that OCA are clinically effective with a high graft survival rate over 20 years. If an OCA graft fails, there is some evidence that revision with a second OCA is also effective, though less so than primary OCA. Economic modelling showed that osteochondral allograft transplantation was highly cost-effective, with costs per quality adjusted life year much lower than many other treatments considered cost effective.

**Conclusions:**

Osteochondral allograft transplantation appears highly cost-effective though the cost per quality adjusted life year varies according to the widely varying costs of allografts. Based on one small study, revision OCA also appears very cost-effective, but more evidence is needed.

**Level of evidence:**

II.

**Electronic supplementary material:**

The online version of this article (10.1007/s00167-019-05392-8) contains supplementary material, which is available to authorized users.

## Introduction

Osteochondral allografts (OCA) replace not only the articular cartilage but also a layer of underlying bone. The articular cartilage is the same thickness as the patient’s own (about 4 mm), and the living chondrocytes are too embedded in the cartilage to trigger a significant immune response. The allograft can, therefore, almost exactly replace a cartilage and bone defect in the host’s knee.

Options are limited for a patient with a defect in both the cartilage and the underlying bone (osteochondral defect), most often due to trauma or osteochondritis dissecans (OCD) [26]. It has been shown in historical studies [41, 63] that patients with OCD, particularly ones where the fragment has been removed, have a very high risk of future osteoarthritis and poor knee function. Most of these patients, as well as those with traumatic lesions, are young and active, and knee arthroplasty is rarely indicated. A total knee arthroplasty (TKA) does not restore full knee function for most young patients. In older patients, a unicompartmental knee arthroplasty may offer slightly better rates of return to sporting activities [16], but knee arthroplasty rarely results in normal knee function. A TKA in a young patient will usually fail in their lifetime, resulting in a need for further arthroplasty. Bayliss et al. [5] reported that the lifetime risk of revision for a TKA was 35% for men and 20% for women having their primary procedure in their early 1950s. Data on the risk of revision for patients younger than this are sparse as it is rarely performed, but the risk of revision is thought to be exponentially higher, due to increased activity as well as longer life expectancy.

Management of the “deep OCD” has been a challenge. Apart from OCA, other options that have been tried include a morcellised bone graft in the base covered with an autologous chondrocyte implantation (ACI) patch (more expensive than OCA), and synthetic grafts. Mosaicplasty can be used to treat osteochondral lesions but donor site morbidity limits this to small lesions. Cartilage restoration techniques such as microfracture and ACI do not replace bone defects and do not do well when the underlying bone is damaged. Some symptoms may be relieved by an unloading osteotomy [46] but this does not resolve the underlying intra-articular damage.

McCulloch et al. [46] have set out the advantages of OCA: the ability to repair larger and deeper defects with mature hyaline cartilage, to resolve the underlying bone defect, and to do so in a single procedure. Briggs et al. [7] note that in the past, OCA had been regarded as a salvage procedure when previous surgery failed, but reported good results in a series of 55 patients who had not had previous surgery. They argue that OCA can be a useful first-line treatment especially in patients with large defects. In their case series, the average defect size was 9.6 cm^2^.

Bugbee et al. [9] provide an overview of OCA in which they note that despite proof of concept evidence going back to the 1980s, there was little use of OCA until the late 1990s, and even then it was carried out mainly in a few specialised centres with local tissue banks.

## Evidence: reviews

Seven recent systematic reviews were identified that covered the use of OCA in the knee, some as part of wider reviews, including other interventions. Quality assessment is reported in the Supplementary file, Table 1.

A review by Salzmann et al. [56] was concerned with the use of particulated juvenile articular cartilage rather than discrete allografts and was omitted. A review by Seow et al. [57] on extracellular matrix and particulate cartilage allografts was also excluded. The Cochrane review by Gracitelli et al. [30] was not included because no studies of OCA were included.

The studies included in the reviews varied, reflecting their different aims. Assenmacher et al. [3] included only studies with a minimum of 9 years of follow-up. Campbell et al. [11] focused on return to sport in athletes and included studies with a minimum follow-up of 12 months, while Krych et al. [35] included studies if they reported return to sport outcome measures. De Caro et al. [17] looked at fresh allografts for large lesions and only included studies with at least ten participants and 1-year follow-up. Rosa et al. [37] were interested in repairs of failed cartilage repair but also reviewed failure rates in the primary repairs. Chahal et al. [12] included studies with a minimum sample size of 10, a minimum follow-up of 12 months and studies that were of allograft transplantation alone or in combination with other techniques including meniscal allograft transplantation and osteotomy.

The conclusions of the reviews are shown in Supplementary file Table 2. Assenmacher et al. [3] reported that 75% of patients had good results at mean of 12.3 years after OCA, with the largest drop in graft survival being at 15–20 years, in patients with mean age 30 years at OCA. De Caro et al. [17] also reported good results, in studies using fresh OC grafts, but identified cost as the main barrier. Chahal et al. [12] also reported good results with fresh or fresh-frozen grafts.

## Evidence: primary studies

Some of the best evidence comes from groups that have built up large cohorts of patients over many years. The supplementary file Table 3 gives summary details of these and other OCA studies.

### Gross et al.

The earliest reports, with longest follow-up, come from the Mount Sinai Hospital, Toronto, group of Allan Gross and colleagues. Their first OCA in the knee was done in 1972. In Gross et al. [31], they report results in femoral condyle and tibial plateau separately, for OCAs done in 1972–1995, with mean follow-up of 10 years. In 60 femoral OCAs, graft survival was 95% at 5 years, 85% at 10 years, and 74% at 15 years. Mean age at OCA was 27 years (range 15–47). In 12 patients, OCA failed, with 9 having TKA. In 65 tibial OCAs, mean age at OCA was 42 (range 26–69) years, and 21 failed and had TKA at mean follow-up of almost 10 years. Graft survival was 95% at 5 years, 80% at 10 years, 65% at 15 years, and 46% at 20 years.

Drexler et al. [19] report results in a subgroup of 27 consecutive patients who had combined distal femoral osteotomy and tibial OCA following failed tibial plateau fracture, from 1981 to 2005. Median age was 41 (range 17–62) years. There were good improvements in clinical scores, and graft survival was 89% at 10 years, 71% at 15 years and 24% at 20 years.

The longest follow-up from the group was by Raz et al. [53], after femoral condyle OCA, with 59% graft survival at 25 years.

### Bugbee et al.

William Bugbee et al. have built up one of the biggest cohorts of people who have had OCA, with over 800 patients. In an overview in 2016 [9], they provide data on results in 527 knees in 467 patients, mean age 34 (range 14–68) having OCA for cartilage injury (35%), OCD (30%), cartilage degeneration (12%), osteonecrosis (8%), and early osteoarthritis (OA) (6%).

Results varied by aetiology and history. The best results were seen in patients who had had osteonecrosis (89% graft survival at mean follow-up 5.6 years, range 2–20 years, and 85% at 12 years) [9, 55] or after previous cartilage injury (98% at 12 years). Good results were also seen after OCA in patients under 18 year of age with 90% graft survival at 10 years, with good improvement in symptom scales [51]. Results were not as good in osteoarthritis (41% at 12 years) and in bipolar injuries (“kissing lesions”) with 46% failures rate in 48 knees [47].

However, for many with OA, the alternative (if they were old enough—many would not be, given mean age 34) would be knee arthroplasty (KA). In those patients, OCA of a femoral hemicondyle might provide at least temporary relief of symptoms pending later Knee arthroplasty, and function would be expected to be better than after knee arthroplasty as the cruciates and menisci (if intact) are retained, meaning knee kinematics and possibly proprioception are preserved.

OCA was largely a salvage procedure in a tertiary centre. Eighty-eight percent of patients had had previous surgery, with an average per patient of two previous procedures. Briggs et al. [7] report that results were better in patients who had not had previous surgery, with OCA survival almost 90% at 5 year and 75% at 10 years, and 61% having some further procedures. Gracitelli et al. [29] reported that OCA after failed previous procedures (including microfracture, mosaicplasty, ACI), in 164 knees, was less successful, with graft survival 82% at 10 years and 75% at 15 years—but still very successful, and accompanied by significant improvements in symptoms. In another study, Gracitelli et al. matched 46 patients who had had previous subchondral bone marrow stimulation procedures with 46 who had OCA as primary procedure [28]. At 10 years of follow-up, graft survival was similar (86% and 87%) but almost twice as many of the prior marrow stimulation group required subsequent procedures (including arthroscopic debridement) as the primary OCA group (44% versus 24%).

Tirico et al. [62] examined results of OCA by size of condylar defect in 156 knees from 1998 to 2014. The average graft area was 6.4 cm^2^, range 2.3–11.5 cm^2^. Most (62%) patients had had OCD. Overall graft survival was 97% at 5 years and 93.5% at 10 years, with no difference by graft size, whether measured as absolute area or relative to knee size. Outcomes were broadly similar but benefits were greater in large defects (> 8 cm^2^).

The size of the cohort allows subgroup analysis. Cameron et al. [10] report the results of 29 OCA grafts of the femoral trochlea alone (1993–2011) with graft survival 100% at 5 years and 92% at 10 years, and good improvements in clinical scores.

Gracitelli et al. [26] report the results of isolated patellar OCA in 28 knees from 1983 to 2010. Results were not as good as in some other sites, with 78% graft survival at 10 years and 56% at 15 years.

Horton et al. [33] report results in 33 patients who had a second OCA after the first failed. At 10 years, 61% of the second OCAs survived, with good symptomatic improvement. The 39% of grafts that failed did so at mean follow-up of 5.5 years.

Nielsen et al. [52] reported a high level of return to sport after OCA, with 79% returning to a high level of performance.

### Cole et al.

Another group with considerable experience is the Rush University group in Chicago, Brian Cole and colleagues. McCulloch et al. [46] concluded that OCA was a safe and effective procedure, in a small group of 25 patients in the years 2000–2003. They had had several previous procedures (mean of 2.3 operations), and represented a tertiary referral group. There was good improvement in Lysholm scores, from 39 to 67 (*p* < 0.0001).

A series of articles from Frank et al. [22–24] reported experience in later years, 2003–2014, in 180 consecutive patients with minimum follow-up of 2 years. Graft survival was 87% at 5 years. There was no difference in failures rates by age—13% in over 40 s, and 16% in under 40 s—or by gender. Concomitant meniscal allograft transplantation (MAT), performed in 36% of patients, caused no problems.

### Williams et al.

Another study comes from the New York Hospital for Special Surgery group, with data prospectively collected by Riley Williams and colleagues from 1999. They have provided a series of papers looking at subgroups, showing that results of OCA are no worse in patients who have had anterior cruciate ligament reconstruction (ACLR) [64] or in those with Body Mass Index (BMIs) over 30 (graft survival 83% at 5 years) [49]. They also found that results in patients aged over 40 (mean age 48, range 40–63 years) were also good, with graft survival 73% at 4 years [65].

Williams et al. [66] have treated elite and other high-performance athletes, and Balaz et al. [4] and Krych et al. [36] have reported high proportions returning to high level performance.

While OCA is regarded as the treatment of choice as a salvage procedure, it is not clear why it was originally regarded by some as only a salvage procedure, given its high success rate. In the Gracitelli et al. study [28] comparing those with and without prior procedures, mean age 27 years, both groups did well, with 11% failure in those with no previous repair attempts and 15% in the previous repair group. By 10 years, survival was no different. Gracitelli et al. [28] attribute this to the technique used in OCA, wherein 3–8 mm of subchondral bone is removed and replaced, including the layer damaged by previous procedures.

Cotter et al. [14] report a series of patients who had had an unsuccessful previous repair attempt after OCD (mostly microfracture, open fixation and loose body removal), and then had OCA. At a mean follow-up of 7.3 years, 82% had returned to sport and were satisfied with the results of surgery. This study was not included in a systematic review by Lamplot et al. [38] of treatment of failed cartilage repairs. Lamplot et al. found three studies of the use of OCA after failed repairs, mainly microfracture, and noted that, unlike with ACI, previous MF did not reduce the success rate of OCA [38].

Return to sport after OCA was also reported by Nielsen et al. [52] in a series of 142 patients, about half of whom were highly competitive athletes, with the rest described as “well-trained and frequently sporting”. 75% returned to sport, including at strenuous levels.

The poorest return to previous activities was reported by Shaha et al. [58] in US soldiers. They found that 42% (16/38) were unable to return to full military duties after OCA, especially if their military activity included combat.

The aims of OCA repair are to eliminate symptoms, restore the normal biomechanics in the knee, and prevent the development of osteoarthritis and the need for knee arthroplasty.

### Historical controls

The most serious limitation in the evidence is the absence of control groups. No RCTs of OCA were found. The studies are mostly before and after studies, which do not give data on the effectiveness of OCA over no, or only non-surgical treatment. It was, therefore, necessary to rely on observational studies of untreated osteochondral or chondral defects, often historical. This is an inherent risk of bias in such comparisons, but it was necessary to make the best use of what data are available.

Messner and Maletius [48] reported progression of OA in 28 athletes with symptomatic chondral defects over a 14-year period, with joint space narrowing.

A considerable proportion of people with osteochondral defects have or had OCD. The natural history of this has been reported in several studies. Linden [41] followed up 67 joints in 58 patients for a mean of 33 years. These patients had had onsets in childhood (mean age 13) or as adults (mean age 29), with 80% of lesions on the medial condyle. Internal fixation was not used, and most had arthrotomy and removal of fragments. The results were different for adult and childhood onsets. At mean follow-up of 33 years, none of the childhood onset cases had severe OA. Of the adult onset cases, over 60% (33/53) had severe OA. The pain of OA came on about 20 years after injury.

Anderson and Pagnani [1] reported that of 19 patients who had OCD fragments removed, 8 had severely abnormal International Knee Documentation Committee (IKDC) scores after as little as 5 years (range 5–20 years). Unlike in the Linden study, no significant differences were seen between those patients whose OCD developed before growth ended. Twyman et al. [63] also reported poor outcomes in a series of 22 patients with onset of OCD before skeletal maturity. After a 34-year follow-up, a third had moderate or severe OA.

A natural history study of articular cartilage defects was carried out by Shelbourne et al. [59]. The defects had been incidental findings in people having ACL reconstruction. Patients with cartilage defects were matched with others having ACL reconstructions but who had no articular cartilage defects. At a mean follow-up of 6 years, there was a little difference in symptom scores. This suggests that OA takes time to develop, but mean defect size in this cohort was only 1.7 cm^2^.

Without OCAs, many of these patients are destined to develop early and severe OA. As noted by Heir et al. [32], some already have considerable impairment in quality of life. Treatment would be by analgesics and rehabilitation such as physiotherapy.

### Survival of OCA grafts

The Assenmacher review [3] summarised mean long-term survival from three studies as

- 5 years = 94%
- 10 years = 84%
- 15 years = 71%
- 20 years = 45%.

Sherman et al. [60] reviewed five studies and reported survivals of 85–100% at 5 years, 71–97% at 10 years, 74–76% at 15 years and up to 66% at 20 years. However, they noted poorer results in people with pre-existing OA, and in patello-femoral lesions.

Both Sherman et al. [60] and Rosa et al. [54] regard OCA as the best option after failure of ACI, microfracture and mosaicplasty.

The longest term study is by Raz et al. [53] with 59% graft survival at 25 years.

Even when OCAs fail, most of the failures occur after a considerable time, such as after 15 years. They can, therefore, postpone knee arthroplasty until an age range where the TKA may be more acceptable to the patient and may not need to be revised. Some patients may have unicompartmental KA first, but which may later be revised to TKA.

## Economic analysis

The knee model starts from the decision to insert OCA. It was assumed that any patients with sufficient malalignment to require osteotomy would have that done first (or at the same time). So the arms of the study are intervention with OCA and non-surgical care.

Failures after OCA arm can be considered for a second OCA, or can go down the same pathway as the no-surgery group. However, most will do well, with over half still successful at 15 years. Some will then fail, but patients may then have reached the age at which knee arthroplasty is acceptable. So the effect of OCA, over a 30-year period, is to avoid KA in many, and to delay it in others. The delay reduces the likelihood of revision TKA being required.

## Modelling

Non-surgical care will include symptomatic relief with analgesics, and may also include physiotherapy. The underlying osteochondral defect will not be affected by these, and patients will progress to osteoarthritis and in due course, knee arthroplasty. If symptoms become severe, they may be considered for earlier than usual knee arthroplasty, with the acceptance that the initial arthroplasty will not last a lifetime, and that subsequent revision(s) will be required.

A major driver in the modelling is knee arthroplasty costs, which depend on the number of arthroplasties per patient per lifetime. Bayliss et al. [42] used the UK Clinical Practice Research Datalink to examine arthroplasty revision rates by age of first arthroplasty. People aged 70 or over at first TKA had only about a 5% chance of needing a revision in their lifetime, but people younger at TKA had a much higher chance, with the highest reported being a 35% revision rate of TKA in men aged 50–54 years. The rate amongst women was about 20% lower. The mean duration in these men from TKA to revision was only about 5 years, meaning that a second or third TKA revision was likely.

The higher revision rate in men may be linked with return to sport. In a systematic review, Witjes et al. [67] found that most of 3261 men had returned to sporting activities 3 months after a TKA. Dagneaux et al. [16] conclude that most people can return to intermediate activities but that return to sport should be gentle and progressive.

So if OCA can avoid revision in most people, or postpone it in others, it can mean that first TKA is at least delayed, and that the need for revision TKA is reduced. For example, if OCA in a 40 year-old can give a good result for 20 years, first TKA at age 60 is much less likely (about 15%) to need to be replaced than a first TKA at age 50 (35%).

The evidence on TKA under age 50 is sparse, and as noted by Lonner et al. [42], most TKA in the under 1950s is done for rheumatoid arthritis (RA), not OA, and so not relevant to this review. (RA is a systemic disease and if someone has severe RA with TKA at, say, age 40, they are likely to have other joints affected and to be physically less active, and unlikely to be taking part in activities that confer a high risk of revision being required.) Lonner et al. reported the results of TKA in 32 patients with OA, who had the procedure under 40 years of age. Good results were seen in 91% (no revision needed) or 87% (either revision or radiological evidence of loosening) at mean follow-up 8 years (range 5–17 years). The TKAs were done from 1982 to 1994. However, the 9% revision rate at 8 years may not be sustained at longer durations.

A proportion, perhaps 30%, will have UKA, because they have single-compartment OA. However, the use of UKA appears to vary regionally and internationally.

### Assumptions for modelling

For survival, the figures from Familiari et al. [21] were used because they are based on a number of studies. (Note that these results are not as good as in some individual studies). Mean survivals:

- 87% at 5 years
- 79% at 10 years
- 73% at 15 years (range 56–84%, five studies)
- 68% at 20 years (range 66–69%, two studies).

One study [53] reported 59% survival at 25 years.

In the base-case, it was assumed that no one has TKA before age 55, so if OCA fails, they will have conservative symptomatic treatment till age 55. In practice, some people may have TKA at age 50, whereas others might postpone it till age 60.

For the no-surgery arm, it was assumed that they have few symptoms for 10 years, on average, because there are two main groups, those with OCD in whom symptoms may not appear for many years, and those with chondral injuries with poor underlying bone structure, who present with pain. They will need non-surgical care till they become eligible for TKA at age 55.

From ages 40 to 55, they will have increasing disutility from OA. By about age 60, at least 60% will have had TKA, whereas by age 60, only at most 22% of the OCA group will have had TKA, assuming that all graft failures do have TKA.

For cost purposes, it was assumed that fresh allografts were used, and that small lesions (under 2 cm^2^) would not receive OCA, but would be treated by, e.g. mosiacplasty [in line with the UK Surgeons Consensus document on ACI and the National Institute for Health and Care Excellence (NICE) guidance on ACI]. In the base-case, the cost of an allograft was taken from the JRF Ortho price list, as £12,850 (http://jrfortho.org/). A lower cost was used in a sensitivity analysis.

### OCA revisions

OCA repairs of osteochondral defects are usually successful, but a proportion fails as reported earlier. After failure of first OCA, it was assumed that a second OCA would be offered, with 10-year survival poorer than after primary, but still around 50% at 10 years. There are few studies on revision OCA.

In the Horton et al. [33] study, all patients had revision of previous OCA. Some other studies include a few patients having revision OCA (Emmerson 5 OCAs [20], Gortz 3 [26], Levy 15 [40], and Meric 3 [47]) after failed primary OCA, but do not give results of these separately, probably because of the small numbers.

So the best evidence on the success of second OCA after failure of the first OCA comes from the Horton study of 33 patients [33]. Failure was defined at progression to KA. The mean age at first OCA was 33 years (range 16–64) with failure in 39% of second OCA at mean 5.5 years. Failure was common in older patients so mean age at failure was 45 years. All of the 13 failures had knee arthroplasty [12 TKA, 1 unicompartmental (UKA)].

It was assumed that revision OCA was less effective than primary OCA, but 61% got good results, and the alternative would have been continuing symptoms and non-surgical care, or arthroplasty at a much younger than ideal age.

Failure of revision OCA was linear over the first 12 years with survival at year 12 about 48%. So each year, approximately 4% fail. After that, Horton et al. [33] report no further failures but numbers by then are very small.

This study, though small and from a centre of excellence, is the best data available currently on revision OCA. The cost-effectiveness was modelled using the same model as for primary OCA, but applying different transition probabilities. However, one problem is what to assume after year 12. One solution is that after year 12, the same failure rates as in primary OCA of about 1.4% per annum can be applied.

An alternative would be to assume no further failures (which is what Horton et al. [33] reported), but that seems over-optimistic.

The results of this modelling must be treated with caution because of the small number of patients reported by Horton et al. [33], but it is the best data available.

The aim of this analysis is to determine whether OCA is cost-effective compared to current standard practice (no OCA), as primary treatment for patients who have a defect both in the cartilage and the underlying bone.

Patients who have had OCA can have a number of outcomes:

- Permanent success—where symptoms are relieved, and no TKA is necessary.
- Failure, in the short term treated symptomatically with analgesics; and in the medium term developing OA treated symptoms with non-surgical care (analgesics and physiotherapy); and in the longer term have a knee arthroplasty.

Assumptions included a mean age at initial osteochondral injury of 30 years, that patients will develop symptomatic osteoarthritis around the age of 40, and might have a knee arthroplasty later, but not until they are aged 55 years or above.

### Model structure

A Markov model was developed within Microsoft Excel^®^ and was considered the most appropriate to determine whether OCA would postpone or avoid knee arthroplasty in the longer term for patients with a defect both in the articular cartilage and the underlying bone. The different health states for the model are shown by the ovals. The model shows all the transitions that can happen between the different health states by the direction of the arrows. The little loop arrows in the left hand corner of the ovals (recurring arrow) means that a patient can stay in that health state for more than one cycle, and perhaps indefinitely, until they die.

**Fig. 1:**
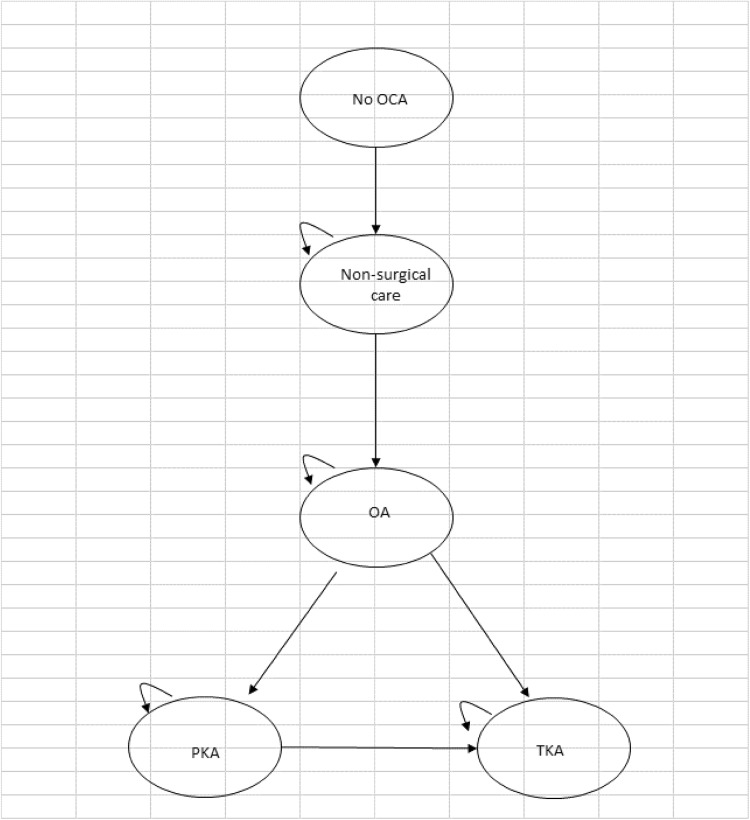
No OCA model structure

Figure 1 shows the model structure for patients who have no OCA (standard care). The starting point of the model is patients aged 30 years. These patients manage their pain with analgesics. When they get to around the age of 40 years, they begin to develop symptomatic OA, which they will manage with a non-surgical care package of analgesics and physiotherapy. When the patient turns 55 years of age, they may choose to have a knee arthroplasty (see Fig. 3). From all health states, patients can die.

**Fig. 2:**
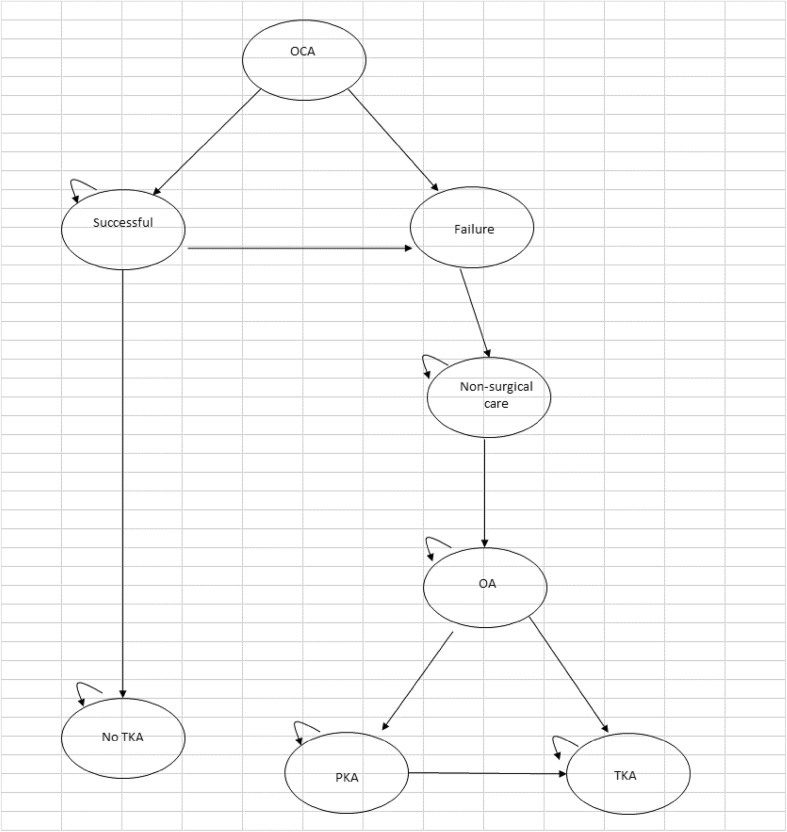
OCA model structure

**Fig. 3:**
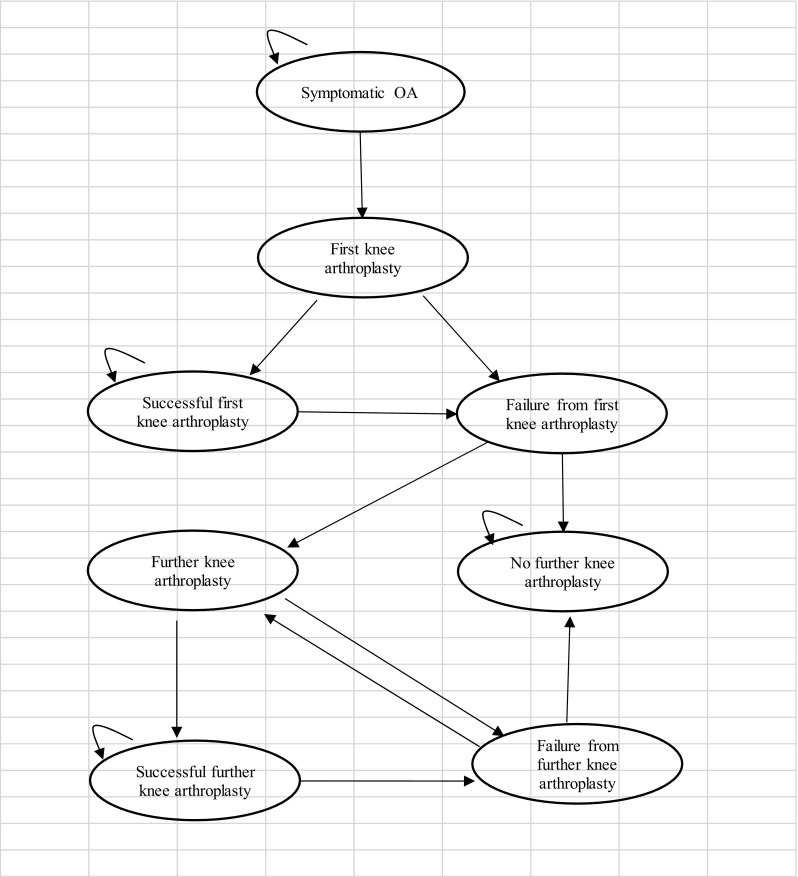
Model structure for knee arthroplasty

Figure 2 shows the model structure for patients who have an OCA transplantation. The starting point of the model is patients aged 30 years who have received an OCA transplantation. After the OCA, patients can then move either to a successful health state where symptoms are relieved or to failure health state where symptoms are not relieved. For those patients who move to the successful health state, some patients can remain there permanently, or over time the OCA can fail and they then move to the failure health state. Patients whose symptoms are not relieved manage their pain with analgesics. When they get to around the age of 40 years they begin to develop symptomatic OA, so they will manage their OA symptoms with a non-surgical care package, which includes analgesics and physiotherapy. When the patient turns 55 years of age, they may choose to have a knee arthroplasty (see Fig. 3). From all health states, patients can die.

Patients over the age of 55 can have a knee arthroplasty or non-surgical care. A patient can move to first knee arthroplasty from the symptomatic OA health state when they reach the knee arthroplasty age range (see Fig. 3). The first knee arthroplasty can be either a UKA or TKA, but all subsequent arthroplasties are assumed to be TKAs. The first knee arthroplasty can be a permanent or temporary success, so the patient moves to the successful first knee arthroplasty health state, or the arthroplasty can fail over time, so they move to the failure of first knee arthroplasty health state, from which patients can choose to have another knee replacement or to have no further knee arthroplasty. The second knee arthroplasty can be a permanent success, or a temporary success, and they move to the failure of further knee arthroplasty health state, from which they can choose to have no further knee arthroplasty and or to have another (third) knee arthroplasty. Patients who move to the no further knee arthroplasty health state choose not to have another knee arthroplasty and stay in this health state. From all health states patients can die. From the knee arthroplasty health states, there is a slight risk of mortality associated with the knee arthroplasty.

## Base-case analysis

For the base-case analysis, the cost-effectiveness of OCA was compared with no OCA using a lifetime horizon (i.e. patients can live to 100 years) with a cycle length for the model set at 1 year and transitions between each health state occurring at the end of each cycle. A hypothetical cohort of 1000 patients with a defect both in the cartilage and the underlying bone with a starting age of 30 years is followed. No differentiation was made by gender. The analysis is conducted from the perspective of the UK National Health Service (NHS) and personal social services (PSS). All costs are in pounds sterling (£) in 2016/2017 prices. Health outcomes are measured in quality-adjusted life years (QALYs), based on their likelihood of the cohort surviving each cycle. Results are expressed as incremental cost per QALY gained. An annual discount rate of 3.5% is applied to both costs and outcomes in line with recommended guidelines.

### Model inputs

#### Transition probabilities

For the base-case analysis, annual transition probabilities were based on data derived from the literature and assumptions from clinical experts. OCA survival (of allograft) was based on rates provided in the systematic review by Familiari et al. [21]. The mean 5, 10, 15 and 20 year survival rates were 86.7%, 78.7%, 72.8% and 67.5%, respectively [21]. Longer term graft survival was based on a study by Raz et al. [53], who reported a 25-year survival rate of 59.0%. These points were fitted onto a graph to check the plausibility of the survival curve and then calculated annual transition probabilities from this survival curve to use in the economic model. Once they move out of the successful health state and into the failure/non-surgical care/OA arm, it was assumed that patient will stay there until they get to the knee arthroplasty age.

For the No OCA arm, patients remain in the non-surgical care/OA arm, until they get to the knee arthroplasty age.

When the patient turns 55 years (bearing in mind that by this point, they would on average be 25 years on from a diagnosis of OCD, so severe OA will be common), it was assumed that 40% will remain in the OA health state, 30% have a UKA and 30% have a TKA.

Transition probabilities for success and failure for patients who needed knee arthroplasties or knee arthroplasty revisions were derived from two studies: Dong and Buxton [18] and Gerlier et al. [25].

### Utilities

For patients who move to the following health states, successful health state or no TKA health state, the utility values for the UK general population were used and adjusted this using an age-related utility decrement [2].

For those patients who move to the failure/non-surgical care health state, a utility value of 0.721 was used based on non-obese patients who had knee pain and were aged between 25 and 44 years from Losina et al. [43]; the authors modelled different pharmacological regimens for knee osteoarthritis prevention.

For patients who developed osteoarthritis, a utility value of 0.645 from Mari et al. [45] was used, which was based on patients who had knee osteoarthritis with a non-pharmacologic option (physical therapy).

Mean utility values are the same for knee arthroplasties after OCA or no OCA and are based on utility values used in our previous report [50]. Before the first knee arthroplasty (UKA or TKA), patients are assumed to have the same utility value (0.615). This value was based on an average of two utility values: (1) the EQ-5D index score at baseline pre-operatively for knee arthroplasty (0.51) [34] and (2) an estimate for TKA operation for knee problem (0.72) [18]. For patients who move to the successful first TKA or UKA health state, a utility value of 0.780 was used [18]. This value was estimated from the generic Knee Society Score scale and was applied to the normal health state after primary TKA. If patients move to the successful further TKA health state, it was assumed that they will have the same utility value as if it was a first TKA. Gerlier et al. [25] was used to obtain two further utility values: (1) for patients for whom TKA has failed, and a further TKA is required, the value was based on the failed TKA/revision health state (0.557) and (2) for patients who move to the no further TKA health state value, this was based on patients who had no clinical success 5 years after surgery (0.691).

### Resource use and costs

All unit costs are presented in pounds sterling (£) in 2016/17 prices. The cost of OCA transplantation includes the costs of the OCA (femoral condyle) graft and the implantation. The implantation cost was based on the costs for major knee procedures for non-trauma patients who are 19 years and older with a CC score 0–1 which was obtained from the NHS reference costs [27]. Before a patient receives the OCA transplantation, they have an outpatient appointment with an orthopaedic consultant. Cost includes three follow-up outpatient clinic visits as most patients are seen between 6 weeks and 3 months post-operation and also eight visits to see a hospital physiotherapist where each session lasts 30 min (see Table 1).

**Table 1:** Base-case mean costs used in the economic model

Resource use	Information	Unit cost (£)	Source
OCA			
Fresh OCA graft	Fresh OCA including implantation (HRG code: HN23C)	£15,560^a^	[27] + expert opinion [27]
Outpatient visit	Consultant-led outpatient first attendance (HRG code: WF01B)	£138.43^a^	[27]
3 post-operation visits	Consultant-led outpatient follow-up attendance (HRG code: WF01A) 8 hospital visits a year (30 min each)	£335.89^a^	[15]
Physiotherapy		£132.00	
Total cost		£16,166.63	
Non-operative package			
Paracetamol	Twice a day per year	£23.21	[8]
Ibuprofen	Once a day per year	£12.47	[8]
Physiotherapy	8 hospital visits a year (30 min each)	£132.00	[15]
Total cost per year		£167.69	
Knee arthroplasty (KA)			
First TKA (UKA or TKA)	Very major knee procedures for non-trauma with CC score 0–1 (HRG code: HN22E)	£5754.17^a^	[27]
Further TKA	Second TKA	£13,551.05^a^	[13]
Outpatient visit	Consultant-led outpatient FU attendance (HRG code: WF01A)	£111.96^a^	[27]

*HRG* Healthcare Resource Group, *CC* complication and comorbidity

^a^Uplifted to 2016/17 prices using the Hospital and Community Health Services (HCHS) index [15]

Patients with OA will receive non-surgical care consisting of analgesics, paracetamol and ibuprofen, and physiotherapy, and eight visits to see a hospital physiotherapist where each session lasts 30 min. Medication costs were obtained from the British National Formulary [8].

The cost for a first knee arthroplasty, either a TKA or a UKA, was obtained from the NHS reference costs [27] using the same assumptions made in our previous work [50]. After a UKA, a second knee arthroplasty would be a TKA, and at a cost of £5754. However, after a TKA, a subsequent TKA is almost double the cost, because the implants are more expensive and it is technically more difficult [13]. Any subsequent knee arthroplasties would all be TKAs at a cost of £13,551. Based on clinical experiences, for the first year after knee arthroplasty (KA), the cost of two outpatient visits was included (see Table 1) [50].

It was assumed that there would be no further costs after the first year if patients enter the successful health states.

### Mortality

Data from the UK general population lifetime tables for age-specific mortality rates (ONS, 2014) were used, combining the average probability of death for men and women. As the cohort ages, mortality rates generally increase throughout the model time horizon and patients can move to the dead state. Patients undergoing surgery for a UKA or TKA are subject to a risk of mortality. To reflect this higher mortality, rates were obtained from a study by Mahomed et al. [44]. For patients undergoing a knee arthroplasty or a knee revision, the mortality rates were reported as 0.7% and 1.1%, respectively [44].

## Results

Table 2 below presents the base-case deterministic results when using an OCA graft price of £12,850. The results highlight even though OCA transplantation is more costly, it is also more effective than not having an OCA. The discounted cost per QALY (incremental cost-effectiveness ratio) is £4692.

**Table 2:** Base-case deterministic cost-effectiveness results

Procedure	Total mean costs	Total mean QALYs	Incremental costs	Incremental QALYs	ICER (cost per QALY gained)
Deterministic—undiscounted					
No OCA	£11,369	37.11	–	–	–
OCA	£23,539	41.51	£12,170	4.40	£2765
Deterministic—discounted					
No OCA	£4828	17.68	–	–	–
OCA	£18,652	20.63	£13,824	2.94	£4692

The key cost driver is the cost of the graft, but over the lifetime horizon, there are QALYs gained from using OCA, and there are cost savings later due to fewer people needing a TKA in the OCA arm.

Table 3 below presents the base-case deterministic results when using an OCA graft price of £3892.50 (€4174) based on costs in Spain. Even though OCA transplantation is slightly more costly, it provided more QALYs than not having an OCA. The discounted incremental cost-effectiveness ratio is £1652.

**Table 3:** Deterministic cost-effectiveness results—changing the cost of the graft

Procedure	Total mean costs	Total mean QALYs	Incremental costs	Incremental QALYs	ICER (cost per QALY gained)
Deterministic—undiscounted					
No OCA	£11,369	37.11	–	–	–
OCA	£14,581	41.51	£3212	4.40	£730
Deterministic—discounted					
No OCA	£4828	17.68	–	–	–
OCA	£9694	20.63	£4867	2.94	£1652

### Sensitivity analyses

Table 4 below presents the deterministic results assuming that if people need a knee arthroplasty they can have it at 45 years instead of 55 years as in our base-case model. This means that they have fewer years of symptoms and hence some QALY gain, but may have a higher TKA revision rate in later years. The results are in line with the base-case model—OCA is more costly but more effective than not having an OCA. The discounted incremental cost-effectiveness ratio is £5084.

**Table 4:** Deterministic cost-effectiveness results—knee arthroplasty at 45 years

Procedure	Total mean costs	Total mean QALYs	Incremental costs	Incremental QALYs	ICER (cost per QALY gained)
Deterministic—undiscounted					
No OCA	£10,891	38.20	–	–	–
OCA	£23,423	42.02	£12,532	3.82	£3283
Deterministic—discounted					
No OCA	£5629	18.24	–	–	–
OCA	£18,910	20.85	£13,282	2.61	£5084

Table 5 below presents the deterministic results for revision OCA using data from Horton et al. [33]. For simplicity and because of the lack of data the cost-effectiveness model was re-run using the probabilities of OCA revision as the primary OCA. Again, results are in line with the base-case model; even though OCA is more costly, it is more effective than not having an OCA. The discounted incremental cost-effectiveness ratio is £6760 (nearly £2000 more than the base-case ICER). However, by generally accepted costs per QALY, this is still very good value. Caveats are required. The study by Horton et al. is small, and comes from one of the world centres of excellence in OCA. But even if the ICER was trebled, it would still fall below the threshold used by NICE in the UK as a guide to value for money.

**Table 5:** Deterministic cost-effectiveness results—survival rates from Horton et al. for second revision

Procedure	Total mean costs	Total mean QALYs	Incremental costs	Incremental QALYs	ICER (cost per QALY gained)
Deterministic—undiscounted					
No OCA	£11,369	37.11	–	–	–
OCA	£25,601	40.18	£14,231	3.07	£4634
Deterministic—discounted					
No OCA	£4828	17.68	–	–	–
OCA	£19,710	19.88	£14,882	2.20	£6760

## Discussion

The results of OCA are generally good. In most cases, there are no other satisfactory options, because most subjects are too young for knee arthroplasty.

In all scenarios, OCA was cost-effective and sensitivity analyses confirmed the robustness of the model. The key cost driver was the cost of the graft but OCA was still cost-effective using the highest price.

The model does have a number of limitations. First, there were no long-term data on utilities, associated with OCA survival or failure. Second, our clinical experience for data on some resources was used in the model, for example, the number of post-operation outpatient visits and for the components of the non-operative package. Practice and, therefore, costs may vary. Finally, no account was taken of any costs to patients such as time off work and loss of pay (productivity).

However, the base-case cost per QALY of £4692 is considerably below the threshold of £20,000 commonly used by NICE, so even if some of the estimates were incorrect and the true ICER was twice that, OCA would still be highly cost-effective.

An arm with metal patches was considered, but it was decided that these were still experimental with insufficient data. A high revision rate was reported with the HemiCAP-Wave patch [39]. However, Becher and Cantillier [6] reviewed five other studies, wherein the revision rate was only about 10%. They reported the results of 169 HemiCAP implants, most successful. However, follow-up KOOS scores were given but not baseline ones, so the amount of benefit cannot be determined.

Data on a more recent device, the second version of the Episealer, are as yet sparse, with two published accounts with ten [61] and two [6] patients. However, such patches may be an option in future once more data are available. One problem with assessing such devices is that they continue to evolve and long-term results may come from superseded versions.

## Conclusions

Osteochondral allograft transplantation appears highly cost-effective.

If a first OCA fails, a second, revision OCA also appears cost-effective, but this is based on only one small study.

## Electronic supplementary material

Below is the link to the electronic supplementary material.

Supplementary material 1 (DOCX 105 KB)
