# Supplementary material for: The cost-effectiveness of osteochondral allograft transplantation in the knee
Source: Knee Surg Sports Traumatol Arthrosc. 2019 Feb 5;27(6):1739–53. doi: 10.1007/s00167-019-05392-8 (PMC6541582; doi:10.1007/s00167-019-05392-8)
Supplement: Supplementary file 1 — Supplementary material 1 (DOCX 105 KB) [file 167_2019_5392_MOESM1_ESM.docx]

KSSTA OCA paper Supplementary File

Table 1: Quality assessment of reviews of osteochondral allografts in knees

| **Review** | **Focused question** | **Eligibility criteria** | **Searches** | **Dual review** | **Validity** | **Study details** | **Publication bias** | **Hetero-geneity** |
| --- | --- | --- | --- | --- | --- | --- | --- | --- |
| Assenmacher 2016[1] | Y | Y | Y | NR | Y | Y | N | NA |
| Campbell et al 2016[10] | Y | Y | Y | Y | Y | Y | N | NA |
| De Caro et al 2015[12] | Y | Y | Y | Y | N | Y | N | NA |
| CADTH 2017[7]^a^ | Y | Y | Y | N | Y | Y | Y | NA |
| Krych 2016[25] | Y | Y | Y | NR | Y | Y | N | Y |
| Chahal et al 2013[11] | Y | Y | Y | Y | Y | Y | N | NA |
| Rosa et al 2017[37] | y | y | Y | y | n | Y | n | NA |

Y, yes; N, no; CD, cannot determine; NA, not applicable; NR, not reported

1. Is the review based on a focused question that is adequately formulated and described?

2. Were eligibility criteria for included and excluded studies predefined and specified?

3. Did the literature search strategy use a comprehensive, systematic approach?

4. Were titles, abstracts, and full-text articles dually and independently reviewed for inclusion and exclusion to minimize bias?

5. Was the quality of each included study rated using a standard method to appraise its internal validity?

6. Were the included studies listed along with important characteristics and results of each study?

7. Was publication bias assessed?

8. Was heterogeneity assessed? (This question applies only to meta-analyses.)

Table 2: Results and conclusions of reviews of OCA in the knee

| **Results** | **Conclusions** |
| --- | --- |
| **Assenmacher et al 2016[1]: Long-term outcomes after osteochondral allograft** | |
| 5 studies included, mean follow-up across studies 12.3 years, range 10 to 17 years. Gross 2005[22] Levy 2013[28] Salai 1997[40] Torga 2006[44] and Drexler 201 [13]  Postoperative Hospital for Special Surgery score mean 84.1.  Mean outcome score improvement:  Knee Society Function Score, 3 studies, 23.1 (95% CI 10.1-36.0, P < 0.01)  Knee Society Knee Score, 2 studies, 26.4 (95% CI, 10.4-42.4, P < 0.01)  Lysholm score, 1 study, 53 (95% CI, 27.4-78.6, P < 0.01)  Mean failure rate at final follow-up 25% (5 studies)  Mean reoperation rate at final follow-up 36% (5 studies)  Mean survival (3 studies) at:  5 years 94%  10 years 84%  15 years 71%  20 years 45% | In most (75%) patients, OCA gave good results at a mean of 12.3 years after surgery. The largest drop in graft survival occurred between 15 and 20 years, in a population with mean age 35 at grafting. Most failures went on to total knee replacements. A few had unilateral KR.  Failure was variably defined, from graft removal or TKA, to Lysholm and KSS scores <70. |
| **Campbell et al 2016[10]: Return to Sport After Articular Cartilage Repair in Athletes’ Knees** | |
| 20 studies (ACI 7; OAT 3, osteochondral allograft 1, microfracture 11), median follow-up 3.6 years (range, 1 to 10.4).  Rate of return to sports:  ACI 84% (p<0.01 vs microfracture)  OAT 89% (p<0.001 vs microfracture)  Osteochondral allograft 88% (p=0.1 vs microfracture)  Microfracture 75%  Average time to return to sports:  ACI 16.0 months  OAT 7.1 months  Osteochondral allograft 9.6 months  Microfracture 8.6 months  Patient-specific factors that influenced outcomes reported (not extracted) | The authors concluded that athletes could return to sports after most interventions, but that microfracture patients were least likely to do so. Only one OCA study was included. (Krych 2012[26] ) |
| **De Caro et al 2015[12]: Large fresh osteochondral allografts of the knee** | |
| 11 studies, mean follow-up 24 months – 13.5 years.  Number of failures range 0-31 (percentages not reported).  General results presented for individual studies only, limited data.  3 studies reported improvement in symptom and/or function scores.  1 study reported survivorship rate: 89% at 5 yr, 82% at 10 yr, 74% at 15 yr, and 66% at 20 yr | Most studies reported good results, some after long follow-up. All but one study used fresh osteochondral allografts. The authors note that no other effective treatment exists at present for large osteochondral lesions. Cost is identified as the main barrier. |
| **CADTH 2017[7]: The Use of Osteochondral Allograft for the Ankle, Knee, and Shoulder: Clinical Effectiveness and Cost-Effectiveness** | |
| Review of reviews | There were four reviews of OCA in the knee, judged by the CADTH team to e of mixed quality. The quality of the primary studies included in those SRs was also judged to be generally poor, and CADTH advise caution in the interpretation of the findings.  The number of primary studies in the knee reviews ranged from one to 19, all case series with no controls. Overall, the review of reviews concluded that OCA reduced pain and improved function in most subjects, and that patient satisfaction was high. A wide range of six to 30 months was reported as the time it took to return to activities or sports.  Graft survival was 91-95% at 5 years, 76-85% at 10 years, and 71-76% at 15 years. The largest drop occurred between 15 and 20 years after the operation. Graft failure (defined as further surgery, including knee replacement and graft removal) occurred in 18-25%.  One problem was that outcomes for a population not receiving osteochondral allografts were not reported.  The authors concluded that prospective RCTs with large samples, longer follow-up, and high quality are needed. |
| **Krych et al 2017**[25]**: Return to sport after the surgical management of articular cartilage lesions in the knee: a meta‑analysis** | |
| 3 allograft studies, follow-up ranged from 24-35 months. Gracitell1 2015[20], McCulloch 2007[29] and Krych 2012[26].  Return to sport was 88%, time to return-to-sports 9.6 (SD 3.0) months | In a wider review of return to sport after cartilage procedures, Krych and colleagues included 3 case series of return to sport after OCA in the knee (including their own 2012 study) with a total of 96 subjects followed up for 24 to 35 months. They concluded that none of the studies provide a high level of evidence. It was notable that the mean defect size was 6.5cm^2^ – OCA was being used for larger lesions. In an earlier broader but non-systematic review [24], Krych et al identified six studies of OAC in the knee (only one of which was included in the return to sport review) and summarised the benefits of OCA as being;   - Both bone and cartilage are replaced - Single operation - The cartilage is hyaline (unlike after microfracture) - Large lesions can be treated.   They regarded that disadvantages as being cost, availability and the possible risk of infection. The six studies reported consistently good results. All used fresh allografts. One series reported SF36 but had only 19 patients (Williams et al [49]). |
| **Chahal et al 2013**[11]**: Outcomes of Osteochondral Allograft Transplantation in the Knee** | |
| 19 studies, mean follow-up 58 months (range 19-120)  Aggregate mean preoperative IKDC score (6 studies) 37.1, postoperative 64.3 (significant in all studies individually).  Aggregate preoperative Lysholm score (4 studies) 39.3, postoperative 70.1 (significant in all studies individually).  Aggregate preoperative Tegner score (3 studies) 3.9, postoperative 5.5 (significant in all studies individually).  No aggregate survivorship reported (2 studies reported separately)  Failure rates 18.1% (review notes differences in definitions and follow-up)  Revisions or removals 14%. Serious failures in 2 patients. | Chahal and colleagues included 19 studies, 17 being retrospective and two prospective, with 644 subjects. The Coleman scores were poor (mean 32, range 19 to 45, so no good quality studies). All studies used fresh or fresh-frozen allografts, with none irradiated. Five studies reported on OA in the knee, finding little or none at follow-up in 65% (72 of 110) of patients.  Clinical outcomes were consistent and favourable.  There was high (86%) satisfaction rate at mean 5 years follow-up. |
| **Rosa et al 2017[37]: How to Manage a Failed Cartilage Repair: A Systematic Literature Review** | |
| 12 studies in allografts, mean follow-up not reported  Studies were not pooled but discussed separately only. | In patients with no previous cartilage repairs, 13-18% failed after OCA, which was less than after microfracture or mosaicplasty. In patients who had had failures of previous procedures, Rosa et al concluded that OCA was “a safe option.” |

I, autologous chondrocyte implantation; OAT, osteochondral autograft transplantation

Table 3: Osteochondral allograft studies

| **Reference** | **Aim** | **Population** | **Study details** | **Key results** |
| --- | --- | --- | --- | --- |
| **Studies from the Scripps Clinic, La Jolla group, Bugbee et al.**  This group set up a database to prospectively collect data and they now have data going back for over 20 years. | | | | |
| Bugbee et al [6] | To provide an overview of OCA in cartilage repair, with a review of results from the La Jolla centre | 527 knees in 467 patients, mean age 34 (range 14 – 68) having OCA for cartilage injury (35%), OCD (30%), cartilage degeneration (12%), osteonecrosis (8%), early OA (6%). 88% of patients had had previous surgery. Femoral condyle lesions large (mean 8cm^2^ , range 1 to 27 cm^2^ ). OCA largely a salvage procedure in a tertiary centre. | Data from prospective clinical database starting 1997. Before and after assessment with D’Aubigne and Postel scale. | The majority of patients improved: 93% less pain, 96% satisfied, 90% would do it again. Success varied by conditions, with best results (86% and over) amongst those with no previous surgery, adolescents, and after osteonecrosis, and poorest (63% good to excellent) in revision OCA.  OCA for OA knee not as effective as in the aforementioned conditions, but can provide an alternative to knee replacement. |
| Briggs 2015 [4]  Conference abstract. | To assess OCA transplantation for cartilage injury in patients with no previous surgical treatment. | OCA transplantation as primary treatment for a chondral or osteochondral defect, any age, no prior surgical treatment of an isolated, Grade III or IV chondral or osteochondral defect, minimum 2-year follow-up. From 1983 onwards.  Commonest problems OCD (44%) and avascular necrosis (31%) | Sample size: 55 (61 knees)  Follow-up: mean 7.6 years (range 1.9-22.6)  Data source: prospective database | Pain and function improved (P < 0.01). OCA survival was 89.5% at 5 years and 74.7% at 10 years. 29.5% had further surgery (11 OCA failures and 7 other surgical procedures). Of the 11 OCA failures, (mean time to failure 3.5 years; range 0.5-13.7), 8 had TKR, 2 had OCA revisions, and 1 had a patellectomy. |
| Cameron 2016[9]  Cameron 2015[8] | To evaluate graft survivorship and clinical outcomes in patients who had OCA to the femoral trochlea. | OCA transplantation of the femoral trochlea alone 1993-2011, age >12 years. | Sample size: 28 (29 knees)  Follow-up: 7.0 years (range 2.1-19.9) | Graft survivorship was 100% at 5 years and 91.7% at 10 years. One patient converted to TKA after 7.6 years.  Mean modified d’Aubigne´-Postel score improved from 13.0 to 16.1, KS-F score from 65.6 to 85.2, and IKDC total score from 38.5 to 71.9; the mean UCLA score was 7.9 postoperatively and KOOS QOL scores improved from 34.0 to 75.1. |
| Emmerson 2017[14] | Hypothesis: Fresh osteochondral allograft transplantation will provide a successful surgical treatment for osteochondritis dissecans  of the femoral condyle. | Patients who had undergone treatment for osteochondritis dissecans of the femoral condyle and had a minimum of 2 years of follow-up, 1980-2003. (Paper also says since 1983, not 1980).  May be some overlap with the patients in the Sadr 1997-2013 group. | Sample size: 64 (66 knees)  Follow-up: mean 7.7 (range 2-22) years. | 15% underwent reoperation. The mean clinical score improved from 13.0 preoperatively to 16.4 postoperatively (P < 0.01). |
| Gracitelli 2015a[20] | To evaluate functional outcomes and survivorship of the grafts among patients who underwent OCA for patellar cartilage injuries. | Patients who had undergone an isolated OCA of the patella between 1983 and 2010. Indications: isolated patellar  lesions with ICRS grades 3 and 4, patients who had failed previous surgical and nonsurgical interventions, and/or who wished to avoid prosthetic arthroplasty | Sample size: 27 (28 knees)  Follow-up: mean 9.7 (7.5) years | 60.7% had further surgery  28.6% were considered OA failures (4 conversions to TKA, 2 conversions to patellofemoral knee arthroplasty, 1 revision OCA, 1 patellectomy). Patellar allografting survivorship was 78.1% at 5 and 10 years and 55.8% at 15 years.  Pain and function improved from the  preoperative visit to latest follow-up. |
| Gracitelli 2015b[21] | To assess the outcome of OCA transplantation as a salvage procedure after various cartilage repair surgeries | Underwent cartilage repair surgery prior to OCA transplantation and minimum follow-up of 2 years. Patients with failed previous SMS, OAT, implantation of synthetic bone plugs, or ACI were included. 1983-2011. | Sample size: 163 (164 knees)  Follow-up: 8.5 years (SD 5.6) | 41.5% of knees had reoperations after OCA transplantation. 18.9% of knees were classified as allograft failures. Median time to failure was 2.6 years (SD 6.8, range 0.7-23.4). Survivorship of the graft was 82% at 10 years and 74.9% at 15 years. Scores on all functional outcomes scales improved significantly from preoperatively to latest follow-up. |
| Gracitelli 2015c[19] | To compare the outcomes of a retrospective matched-pair cohort of (1) primary OCA transplantation and (2) OCA transplantation after failure of previous SMS. | Consecutive series with OCA as a primary treatment, (group 1), matched to a non-consecseries that underwent OCA transplantation after failure of previous SMS (group 2). Minimum follow-up of 2 years. 1983-2011. | Sample size: 92 (group 1 46, group 2 46) knees  Follow-up: group 1 7.8 (SD 5.1 years), group 2: 11.3 (SD 6.6 years) | 24% in group 1 had reoperations, compared with 44% in group 2 (P = 0.04). The OCA was classified as a failure in 11% of knees and 15% of knees) in group 2 (P = 0.53). At 10 years of follow-up,  survivorship of the graft was 87.4% and 86% in groups 1 and 2, respectively. Both groups showed improvement in pain and function on all subjective scores from preoperatively to the latest follow-up (all P <0.001). |
| Görtz 2010[18] | To ask if fresh OCAs would (1) heal to host bone in the presence of osteonecrosis, (2) provide a clinically meaningful decrease in pain and improvement in function, and (3) prevent or postpone the need for arthroplasty. | Corticosteroid-associated osteonecrosis, age <50 years (1984-2006) | Sample size: 22 (28 knees)  Follow-up: mean 67 months, range 25–235 | 5 knees failed.  Graft survival rate 89%  Mean D’Aubigne´ and Postel score  improved from 11.3 to 15.8; 76% had a score > 15.  Mean IKDC pain score improved from  7.1 to 2.0, mean IKDC function score from 3.5 to 8.3, and mean Knee Society function score from 60.0 to 85.7. |
| Horton 2013[23] | To evaluate outcomes of patients who have undergone revision osteochondral allograft transplantation of the knee. | Revision OCA in the knee, ≥ 2 years from surgery, and minimum 2 years’ follow-up. 1983-2012. | Sample size: 33  Follow-up: mean 10 years (range 2.4-26) for those with grafts surviving | 39% had failed results after revision OCA transplantation, mean time to failure 5.5 years. The remaining 61% had surviving revision allografts, mean graft survival 10 years. Mean pain and function scores at the last follow-up were improved. |
| Levy 2013[28] [28] | To determine (1) pain and function, (2) frequency and types of reoperations, (3) survivorship, and (4) predictors of OCA failure | OCA of the femoral condyle (1983-2001). Indications: presence of a painful chondral or osteochondral lesion(s) of the femoral condyle and failure of previous nonsurgical or surgical treatments. | Sample size: 122 (129 knees)  Follow-up: median 13.5 years, (range 2.4-27.5) | Mean modified Merle d’Aubigne´-Postel score improved from 12.1 to 16, mean IKDC pain score from 7.0 to 3.8, mean IKDC function score from 3.4 to 7.2, and mean KS-F score from 65.6 to 82.5.  47% of knees underwent reoperations.  24% of knees failed at a mean of 7.2 years.  10 year survivorship 82%  15 year survivorship 74%  20 year survivorship 66% |
| Meric 2015[30] | To evaluate the outcomes of patients who had undergone OCA transplantation for reciprocal bipolar cartilage injuries (‘‘kissing lesions’’) of the knee | OCA transplantation for bipolar cartilage lesions of the knee from 1983 to 2010. Indications: reciprocal lesions in the patellofemoral joint and tibiofemoral joint, ICRS grades 3 and 4, failed previous surgical and nonsurgical interventions and/or wished to avoid prosthetic arthroplasty. | Sample size: 46 (48 knees)  Follow-up: 7 years (range 2.0-19.7). | Survivorship of the bipolar OCA was 64.1% at 5 years. 63% of knees underwent further surgery; 46% were considered failures (3 OCA revisions, 14 total knee arthroplasties, 2 unicondylar arthroplasties, 2 arthrodeses, and 1 patellectomy). Mean modified Merle d’Aubigne´-Postel score improved from 12.1 to 16.1; 88% of surviving allografts scored ≥15. Mean IKDC pain score improved from 7.5 to 4.7, mean IKDC function score improved from 3.4 to 7.0. Mean KS-F score improved from 70.5 to 84.1. |
| Murphy 2014[31]  Earlier report Pennock 2013 (abstract only)[35] | To describe a 28-year experience with OCA transplantation in patients younger than 18 years with a focus on subjective outcome measures, return to activities, and allograft survivorship. | Paediatric and adolescent patients with fresh OCA transplantation in knee; <18 years at time of surgery and ≥ 2 years past date of index surgery. 1983 onwards.  Aetiologies: osteochondritis dissecans, avascular necrosis, traumatic chondral injury, degenerative chondral lesion and fracture. | Sample size: 39 (43 knees)  Follow-up: mean 8.4 years (range 1.7-27.1) | 11.6% knees experienced clinical failure at median of 2.7 years (range, 1.0-14.7). Four failures were salvaged successfully with another OCA transplant. One patient underwent prosthetic arthroplasty 8.6 years after revision allograft. Graft survivorship was 90% at 10 years. Of the knees with grafts in situ, 88% rated good/excellent (18-point scale). Mean IKDC improved from 42 preoperatively to 75 postoperatively, and Knee Society function score improved from 69 to 89 (both P<0.05). |
| Nielsen 2017[32] | To determine if athletic patients undergoing OCA transplantation returned to sport, assess reasons for not returning to sport, and ascertain patient and graft-related characteristics that differed between those who returned or did not return to sport. The secondary aims were to assess graft survivorship and patient-reported subjective outcome measures (pain, function, satisfaction) among athletic patients undergoing OA transplantation. | Primary OCA transplantation by single surgeon 1998-2014, participated in sport or recreational activity before the cartilage injury, and did  not undergo major concomitant surgery (osteotomy, anterior  cruciate ligament [ACL] reconstruction, or meniscal  allograft) at the time of OCA transplantation. | Sample size: 142 (149 knees)  Follow-up: 6 years (range 1.0-15.8) | 75.2% of knees returned to sport or recreational activity.  79% were able to participate in a high level of activity (moderate, strenuous, or very strenuous) postoperatively. After OCA transplantation, 25.5% of knees underwent further surgery; 14 knees (9.4% of entire cohort) were considered allograft failures. Among the 135 knees that had the graft remaining in situ, pain and function improved from preoperatively to the latest follow-up on all measures. |
| Sadr 2016[39] Earlier abstract Sadr 2014 [38] | to determine the clinical outcome of a large cohort of patients (juvenile and adult) who received fresh OCA transplantation for the surgical management of osteochondritis dissecans (OCD) after failure of other treatments. | Patients who had undergone OCA transplantation for OCD (type III or IV) by a single surgeon, 1997-2013, minimum 2 years follow-up.  The 2014 abstract reported results from an earlier but overlapping period, 1983-2010, with 181 knees in 164 patients. | Sample size: 135 (149 knees)  Follow-up: 6.3 years (range 1.9-16.8) | 23% had reoperations, of which 8% were classified as failures (7 OCA revisions, 3 unicompartmental knee arthroplasties, and 2 total knee arthroplasties). OCA survivorship was 95% at 5 years and 93% at 10 years. Of the 137 knees whose grafts were still in situ at the latest follow-up, the mean modified Merle d’Aubigne´ and Postel score was 16.8; IKDC pain, function, and total scores were 2.1, 8.1, and 82.3; and KS-F and KS-K scores were 95.7 and 94.3, respectively.  In the earlier period 31% had re-operations with 13% classed as failures. |
| Schmidt 2017[41] | To investigate the relationship between prolonged fresh graft storage and clinical outcomes of OCA transplantation. | Patients who received ‘‘early release’’ grafts 1997-2002 (mean storage time 6.3 days, range 1-14) or ‘‘late release’’ grafts 2002-2008 (mean storage time 20.0 days range 16-28). Minimum follow-up of 2 years | Sample size: 150 (75 early release, 75 late release)  Follow-up: early release: 11.9 years (range 2.0-16.8), late release: 7.8  years (range 2.3-11.1) | Failure occurred in 25.3% of the  early release group and 12.0% of the late release group (P = 0.036). Median time to failure 3.5 years (range 1.7-13.8) and 2.7 years (range 0.3-11.1) for the early and late release groups, respectively. The 5-year survivorship of OCAs was 85% for the early release group and 90% for the late release group (P = 0.321). No differences in postoperative pain and function between the groups. |
| Tirico 2018  [43] | The aim of this study was to assess success of OCA by size of defect. | Patients who had OCA from 1998 to 2014 for isolated lesions of a femoral condyle. 62% had OCD. | 156 knees in 143 patients. Mean age 29.6, 63% male. Mean graft area 6.4 cm^2^, range 2.3 to 11.5 cm^2^. Mean follow-up 6 years. | Overall graft survival was 97% at 5 years and 93.5% at 10 years, with no difference by graft size, whether measured as absolute area or relative to knee size. Outcomes were broadly similar but benefits were greater in large defects (>8 cm2). |
| **Studies from the Chicago group, Brian Cole and colleagues. This is another group that has built up a prospective database.** | | | | |
| McCulloch 2007 [29] | To assess results of prolonged fresh OCA grafting, stored for up to 42 days. | 25 consecutive patients having fresh prolonged storage OCA grafts for resurfacing of full-thickness cartilage defects of at least 2 cm^2^ in the femoral condyle. Mean age 35, range 17-49. 72% male. Mainly (68%) medial condyle. 96% had had previous procedures, including 18 meniscectomies and 11 microfractures. Mean number of prior procedures (excluding diagnostic arthroscopy) was 2.3 and they were mostly a tertiary referral group.  Concomitant procedures 60%: meniscal transplantations, opening wedge high tibial osteotomies, and removal of previous osteotomy plate. | Minimum follow-up 2 years, mean 35 months, range 24 to 67 months. Concomitant procedures 10 MAT and 4 HTOs. Mean age 35 (range 17 to 49). Years 2000 to 2003 | Improvements in Lysholm (39 to 67), IKDC (29 to 58), and all KOOS components, including significant improvements in KOOS QoL at 2.9 years follow-up. Statistically significant improvements in SF-12 physical component but not SF-12 mental component.  88% of grafts incorporated into host bone.  Little difference in results between OCA alone and OCA + MAT groups.  At 2.9 years follow-up, 8% had failure secondary to allograft fragmentation (allograft removal followed by a microfracture) or marked pain for more than 6 months.  Conclusion: prolonged storage is safe in OCA grafting. |
| Frank 2017[17]  Frank 2018[15]  Frank 2018[16] | To assess survival for OCA transplantation and report findings at reoperations  To compare results for male and female patients under and over 40 years of age. | Consecutive patients undergoing primary OCA transplant by a single surgeon 2003-2014, with minimum follow-up 2 years. Included if they had undergone prior ipsilateral knee surgery (other than prior OCA)  or concomitant procedures  (including MAT, ligament reconstruction, and/or corrective realignment procedures).  36% had MAT at same time as OCA. | Sample size: 180  Follow-up: 5.0 (SD 2.7 years) | 37% had reoperation at a mean of 2.5 (SD 2.5 years). 87% allograft survival at mean 5 years after OCA. Failures in 13% at a mean of 3.6 (SD 2.6) years, defined as revision OCA transplant (n = 7), conversion to arthroplasty (n = 12), or arthroscopic appearance of a poorly incorporated allograft (n = 5).  Excluding the failed patients, statistically and clinically significant improvements were found in the Lysholm score, IKDC score, KOOS, and SF–12 PCS at final follow-up (P < .001 for all). Patients who needed reoperation also improved but less so.  No differences by age in reoperation rate, time to reoperation, or failure rate (>40 years: 13%; ≥40 years: 16%). No significant differences in number of complications, outcome scores, or time to failure between the sexes.  Concomitant MAT caused no problems. |
| **Studies from the Mount Sinai Hospital, Toronto group, Allan Gross and colleagues** | | | | |
| Gross 2005[22]  Abstract: Aubin et al 2001 [2] | To examine the long term clinical and radiological results as well as the survivorship of fresh OCAs for post-traumatic defects around the knee.  The long-term clinical outcomes and survival analysis are presented for patients a minimum of 5 years from OA transplant surgery of the medial or lateral femoral condyle for post-traumatic unipolar defects. | Traumatic unipolar osteochondral defects of at least 3 cm in diameter and 1 cm deep, age <60 years (1972-1995) | Sample size: femoral condylar grafts 60; tibial plateau grafts 65  Follow-up: femoral condylar 120 months, range 58–259;  tibial plateau 11.8 years, range 2–24  Data collection: prospectively collected database | Femoral condylar grafts:  Graft failures: 12 (3 removal, 9 total knee replacement)  5 year survivorship 95%  10 year survivorship 85%  15 year survivorship 74%  Of those with surviving grafts, HSS was 83 points. Transplant to medial or lateral condyle had no bearing on long term outcomes. Of 38 with radiographs, 48% had no or mild arthritis, 26% had moderate and 26% had severe arthritis.  Tibial plateau grafts:  Conversion to TKA: 21  5 year survival 95%  10 year 80%  15 year 65%  20 year 46% |
| Drexler 2015[13] | To examine the long-term survivorship and functional outcome of distal femoral varus osteotomy with focal OCA following failed lateral tibial plateau fracture surgery | Consecutive series (1981-2005) of distal femoral varus osteotomies combined with focal OCA. All had previous open reduction with internal fixation surgery of a lateral tibial plateau fracture, with continued lateral knee pain. Median age at OCA 41 years, range 17-62. | Sample size: 27  Follow-up: median 13.3 years, range 2–31  Data collection: medical records and database | KSKS increased from median 54.6 to 83.8 points at 2 years (p<0.01), still at end of follow-up.  KSKS increased from medial 50.6 to 71.1 at 2 years (p<0.01), still significant at end of follow-up.  10 year survivorship 88.9% (± 4.6)  15 year survivorship 71.4% (± 18.1)  20 year survivorship 23.8% (± 11.1) |
| Raz 2014[36] | To examine the long-term survival and clinical outcomes of fresh OCA for posttraumatic and osteochondritis dissecans  defects in the knee. | Unipolar OCA transplant to the femoral condyle >15 years from time of search, 1972-1995. Age <50 years at surgery, presented with a posttraumatic osteochondral or osteochondritis dissecans defect limited to the distal aspect of the femur (unipolar) and was larger than 3 cm in diameter and 1 cm in depth. | Sample size: 58  Follow-up: mean 21.8 years (range 15- 32)  Data collection: database | 13/58 (22.4%) required further surgery; 3 underwent graft removal, 9 converted to TKA, 1 underwent multiple debridements followed by above-the-knee amputation. Three died due to unrelated causes. Kaplan-Meier analysis of graft survival showed rates of 91%, 84%, 69%, and 59% at 10, 15, 20, and 25  years, respectively. Patients with surviving grafts had good function, with a mean modified HSS score of 86 at 15 years or more. |
| **Articles from the New York Hospital for Special Surgery group. These papers were based on data from a prospective registry started in 1999 by R J Williams.** | | | | |
| Krych 2012[26]  Possible partial overlap with Balazs 2018[3]. | To review the rate of return to athletic activity after OCA transplantation in the knee and to identify any potential risk factors for not returning to sport. No details of which sports.  Balaz 2018 basketball only. | Patients who regularly participated in sports before articular cartilage injury with isolated chondral and osteochondral lesions of the knee, ≥2.5 cm^2^ and without generalized osteoarthritis, age 18-50 years (2000-2010). Fresh OCA.  Balaz reported results in 11 basketball players who had full-thickness cartilage injuries, four professional and 7 college players. 14 treated lesions, mainly lateral condyle and trochlea. Mean defect size 5.1 cm^2^. | Sample size: 43  Follow-up: mean 2.5 years (rage 1-11)  Data collection: registry | Limited return to sport possible: 88%  Return to pre-injury level: 79% (time to return 9.6 (SD 3.0) months).  Balaz reported 80% return to sport with no reduction in performance level, after mean 14 months (range 6 to 26 months). |
| Wang 2017[46] | to compare the clinical outcomes of patients treated with non-orthotopic (lateral-to-medial condyle or medial-to-lateral condyle) OCA with those treated with traditional orthotopic (medial-to-medial condyle or lateral-to-lateral condyle) OCA. | Inclusion criteria: skeletal maturity; symptomatic focal cartilage lesions of the medial or lateral femoral condyle classified as Outerbridge grade III or IV at the time of arthroscopic surgery and not involving substantial bone loss requiring additional bone-grafting; treatment with fresh OCA; minimum 2 years follow-up. | Sample size: 77 (orthotopic 50, non-orthotopic 27)  Follow-up: orthotopic 4.4 years (range 2-16), non-orthotopic 3.4 years (range 2-11)  Data source: registry | Reoperation (p = 0.427) and failure (p = 0.917) rates did not differ  significantly between groups. Both groups demonstrated significant increases in the SF-36 physical functioning and pain, IKDC, and Knee Outcome Survey-Activities of Daily Living (KOS-ADL) scores compared with baseline (p < 0.004). Outcome scores (baseline and postoperative) and change scores did not differ significantly between groups. |
| Wang 2017 [45] | Aim: to compare outcomes of OCA in patients who had had ACL reconstruction with those with intact ACLs. Hypothesis: ACLR does not normalise knee kinematics so OCA may be more likely to fail. |  | 50 ACL intact and 25 ACLR. Minimum follow-up 2 years, mean 3.9 years, range 2-14). Mean age 36 | % year OCA graft survival 79% with intact ACL and 85% with reconstructed. So OCA is not less successful in patients with ACLR. |
| Wang 2018 [47] | OCA in patients aged over 40. |  | 51 patients aged 40-63, mean 48 years. 14 (27%) had had previous repair attempts (mainly MF) and 12 had had previous ACLR. | 14 (27%) OCA failure at 4 years, one revision OCA, 5 UKR, 8 TKR. Higher failure rates with more prior surgery and baseline OA KL of 2 or more. Failure rate higher than in younger populations from other studies, but still 73% graft survival at 4 years. 88% at 2 years |
| Wang 2018[48] | OCA in patients with BMI >30. 2000 to 2015 | 74% had had previous surgery.  Mean BMI 33, range 30-39. | 31 patients mean age 35. Mean BMI 33, range 30-39. | 5 year OCA graft survival 83%. Substantial symptomatic improvement. So BMI > 30 should not rule out OCA. |
| **Other studies** | | | | |
| Brown et al, 2011[5]  Portland, Oregon USA | OCA transplant to repair grade 4 International Cartilage Repair Society articular cartilage defects of the femoral condyle, 2006-2008 | Aetiology: OCD (11), focal OA (23), avascular necrosis (2). Nine (26%) had concomitant procedures including ACL reconstruction, tibial osteotomy, medial patellofemoral ligament reconstruction/ lateral release, meniscus transplant. | Follow-up: 2 years  Sample size: 34 (45 grafts).  Average lesion size 5.7 cm^2,^, range 1.5-15 cm^2^. | Significant improvement in pain and sports/recreation function, but not in symptoms or activities of daily living.  Significant improvements in KOOS QoL at 2 years. IKDC improved from 45 to 62. One patient required TKR after 2 years. |
| LaPrade et al 2009[27]  Minnesota | Main indication was presence of a symptomatic full-thickness articular cartilage defect of >3 cm^2^ on the femoral condyles. Consecutive cases from 2002. All grafts were refrigerated. | 23 patients. Mean age 31 (16 to 47) years. 57% male. 83% medial condyle. 17.4% had additional procedures including tibial osteotomy if required, and patients with >50% loss of the meniscus in the affected compartment had concurrent MAT. | Follow-up mean 3 years, range 1.9 to 4 yrs. | Statistically significant improvements on CKRS overall and individual component scores. IKDC improved from 52 to 68 (p < 0.03) |
| Pearsall et al, 2011[33]  University of South Alabama, USA  Earlier study, Pearsall et al 2008 [34] may have some patients in common | Aim: to compare success of OCA with refrigerated and frozen allografts. “Fresh” allografts defined as harvest within 24 hours of donor’s death and time from harvest to implant 7 days or less. | Tegner 3 or greater activity level; articular cartilage damage limited to 1 or 2 compartments; biomechanical knee alignment that was less than 5° of varus or valgus or correctable with a distal femoral or proximal tibial osteotomy; and failure of conservative measures including non-steroidal anti-inflammatory medications and physical therapy for a minimum of 3 months. Not explicitly reported but a proportion had tibial or distal femoral osteotomies. | Age : 48 (17-69)  % male: 68.8  Follow-up: average 46 months (range 24-60)  Sample size: 26  1998-2002 18 refrigerated and 9 frozen grafts. (2008 paper had 12 and 12) | Six failures (all refrigerated) had KR. 76% survival at 4 years. Mean WOMAC score improved from 46 to 66, and KSS from 104 to 132.  Statistically significant improvements in the Knee Society Score (KSS)  The paper mentions allograft “plugs” so may have used a mosaicplasty technique rather than single large OCAs. |
| Shaha 2013[42]  Hawaii | To assess the ability of an active-duty military population to return to a preinjury level of duty/activity after treatment of a large chondral defect with OCA transplantation | Active-duty military population who underwent OCA (2002-2011). Indication: symptoms were sufficient to limit their activity and ability to function in their occupational role and they had failed to improve with non-operative management | Sample size: 38  Follow-up: mean 4.1 years (range 0.6-8.9)  Data collection: database | Overall rate of return to full duty: 28.9%  Return to limited activity: 28.9%  Unable to return to military activity: 42.1%  Return to pre-injury level of sport: 5.3%  These results are much poorer than in most case series. |

ACI: autologous chondrocyte implantation; ICRS: International Cartilage Repair Society; KS-F, Knee Society Function score; OCA: Osteochondral Allograft; OA osteoarthritis; OAT: osteochondral autograft transplantation; SMS: subchondral marrow stimulation; TKA: Total knee arthroplasty; UCLA: University of California, Los Angeles activity score; UKA: Unicompartmental knee arthroplasty

References

1. Assenmacher AT, Pareek A, Reardon PJ, Macalena JA, Stuart MJ, Krych AJ (2016) Long-term Outcomes After Osteochondral Allograft: A Systematic Review at Long-term Follow-up of 12.3 Years. Arthroscopy 32:2160-2168

2. Aubin PP, Cheah HK, Davis AM, Gross AE (2001) Long-term followup of fresh femoral osteochondral allografts for posttraumatic knee defects. Clinical Orthopaedics & Related Research S318-327

3. Balazs GC, Wang D, Burge AJ, Sinatro AL, Wong AC, Williams RJ (2018) Return to Play Among Elite Basketball Players After Osteochondral Allograft Transplantation of Full-Thickness Cartilage Lesions. Orthopaedic Journal of Sports Medicine 6:7

4. Briggs D, Sadr KN, Pulido P, Bugbee W (2015) The use of osteochondral allograft transplantation for primary treatment of cartilage injuries in the knee. Orthopaedic Journal of Sports Medicine 3 Supplement 1:

5. Brown D, Shirzad K, Lavigne SA, Crawford DC (2011) Osseous Integration after Fresh Osteochondral Allograft Transplantation to the Distal Femur: A Prospective Evaluation Using Computed Tomography. Cartilage 2:337-345

6. Bugbee WD, Pallante-Kichura AL, Gortz S, Amiel D, Sah R (2016) Osteochondral allograft transplantation in cartilage repair: Graft storage paradigm, translational models, and clinical applications. Journal of Orthopaedic Research 34:31-38

7. CADTH. *The Use of Osteochondral Allograft for the Ankle, Knee, and Shoulder: Clinical Effectiveness and Cost-Effectiveness.* Ottawa (ON): Canadian Agency for Drugs and Technologies in Health;2017.

8. Cameron JI, Pulido P, DeYoung A, Gortz S, Bugbee W (2015) Outcome following isolated osteochondral allograft transplantation of the femoral trochlea. Orthopaedic Journal of Sports Medicine 3:

9. Cameron JI, Pulido PA, McCauley JC, Bugbee WD (2016) Osteochondral Allograft Transplantation of the Femoral Trochlea. American Journal of Sports Medicine 44:633-638

10. Campbell AB, Pineda M, Harris JD, Flanigan DC (2016) Return to Sport After Articular Cartilage Repair in Athletes' Knees: A Systematic Review. Arthroscopy 32:651-668.e651

11. Chahal J, Gross AE, Gross C, Mall N, Dwyer T, Chahal A, et al. (2013) Outcomes of osteochondral allograft transplantation in the knee. Arthroscopy 29:575-588

12. De Caro F, Bisicchia S, Amendola A, Ding L (2015) Large fresh osteochondral allografts of the knee: a systematic clinical and basic science review of the literature. Arthroscopy 31:757-765

13. Drexler M, Gross A, Dwyer T, Safir O, Backstein D, Chaudhry H, et al. (2015) Distal femoral varus osteotomy combined with tibial plateau fresh osteochondral allograft for post-traumatic osteoarthritis of the knee. Knee Surgery, Sports Traumatology, Arthroscopy 23:1317-1323

14. Emmerson BC, Gortz S, Jamali AA, Chung C, Amiel D, Bugbee WD (2007) Fresh osteochondral allografting in the treatment of osteochondritis dissecans of the femoral condyle. Am J Sports Med 35:907-914

15. Frank RM, Cotter EJ, Lee S, Poland S, Cole BJ (2018) Do Outcomes of Osteochondral Allograft Transplantation Differ Based on Age and Sex? A Comparative Matched Group Analysis. Am J Sports Med 46:181-191

16. Frank RM, Lee S, Cotter EJ, Hannon CP, Leroux T, Cole BJ (2018) Outcomes of Osteochondral Allograft Transplantation With and Without Concomitant Meniscus Allograft Transplantation: A Comparative Matched Group Analysis. Am J Sports Med;10.1177/0363546517744202363546517744202

17. Frank RM, Lee S, Levy D, Poland S, Smith M, Scalise N, et al. (2017) Osteochondral Allograft Transplantation of the Knee: Analysis of Failures at 5 Years. American Journal of Sports Medicine 45:864-874

18. Gortz S, De Young AJ, Bugbee WD (2010) Fresh osteochondral allografting for steroid-associated osteonecrosis of the femoral condyles. Clinical Orthopaedics & Related Research 468:1269-1278

19. Gracitelli GC, Meric G, Briggs DT, Pulido PA, McCauley JC, Belloti JC, et al. (2015) Fresh osteochondral allografts in the knee: comparison of primary transplantation versus transplantation after failure of previous subchondral marrow stimulation. Am J Sports Med 43:885-891

20. Gracitelli GC, Meric G, Pulido PA, Gortz S, De Young AJ, Bugbee WD (2015) Fresh osteochondral allograft transplantation for isolated patellar cartilage injury. American Journal of Sports Medicine 43:879-884

21. Gracitelli GC, Meric G, Pulido PA, McCauley JC, Bugbee WD (2015) Osteochondral Allograft Transplantation for Knee Lesions after Failure of Cartilage Repair Surgery. Cartilage 6:98-105

22. Gross AE, Shasha N, Aubin P (2005) Long-term followup of the use of fresh osteochondral allografts for posttraumatic knee defects. Clinical Orthopaedics & Related Research 79-87

23. Horton MT, Pulido PA, McCauley JC, Bugbee WD (2013) Revision osteochondral allograft transplantations: do they work? American Journal of Sports Medicine 41:2507-2511

24. Krych AJ, Gobbi A, Lattermann C, Nakamura N (2016) Articular cartilage solutions for the knee: present challenges and future direction. Journal of ISAKOS: Joint Disorders &amp; Orthopaedic Sports Medicine;10.1136/jisakos-2015-000037

25. Krych AJ, Pareek A, King AH, Johnson NR, Stuart MJ, Williams RJ (2017) Return to sport after the surgical management of articular cartilage lesions in the knee: a meta-analysis. Knee Surgery, Sports Traumatology, Arthroscopy 25:3186-3196

26. Krych AJ, Robertson CM, Williams RJ, 3rd, Cartilage Study G (2012) Return to athletic activity after osteochondral allograft transplantation in the knee. American Journal of Sports Medicine 40:1053-1059

27. LaPrade RF, Botker J, Herzog M, Agel J (2009) Refrigerated osteoarticular allografts to treat articular cartilage defects of the femoral condyles. A prospective outcomes study. J Bone Joint Surg Am 91:805-811

28. Levy YD, Gortz S, Pulido PA, McCauley JC, Bugbee WD (2013) Do fresh osteochondral allografts successfully treat femoral condyle lesions? Knee. Clinical Orthopaedics and Related Research 471:231-237

29. McCulloch PC, Kang RW, Sobhy MH, Hayden JK, Cole BJ (2007) Prospective evaluation of prolonged fresh osteochondral allograft transplantation of the femoral condyle: minimum 2-year follow-up. Am J Sports Med 35:411-420

30. Meric G, Gracitelli GC, Gortz S, De Young AJ, Bugbee WD (2015) Fresh osteochondral allograft transplantation for bipolar reciprocal osteochondral lesions of the knee. American Journal of Sports Medicine 43:709-714

31. Murphy RT, Pennock AT, Bugbee WD (2014) Osteochondral allograft transplantation of the knee in the pediatric and adolescent population. American Journal of Sports Medicine 42:635-640

32. Nielsen ES, McCauley JC, Pulido PA, Bugbee WD (2017) Return to Sport and Recreational Activity After Osteochondral Allograft Transplantation in the Knee. American Journal of Sports Medicine 45:1608-1614

33. Pearsall AW, Madanagopal SG, Tucker J (2011 ) The Evaluation of Refrigerated and Frozen Osteochondral Allografts in the Knee. Surgical Science 2:232-241

34. Pearsall AWt, Madanagopal SG, Hughey JT (2008) Osteoarticular autograft and allograft transplantation of the knee: 3 year follow-up. Orthopedics 31:73

35. Pennock AT, Murphy RT, Bugbee W (2013) Osteochondral allografting for knee lesions in the pediatric and adolescent population. Orthopaedic Journal of Sports Medicine 1:

36. Raz G, Safir OA, Backstein DJ, Lee PT, Gross AE (2014) Distal Femoral Fresh Osteochondral Allografts: Follow-up at a Mean of Twenty-two Years. Journal of Bone & Joint Surgery - American Volume 96:1101-1107

37. Rosa D, Di Donato SL, Balato G, D'Addona A, Smeraglia F, Correra G, et al. (2017) How to Manage a Failed Cartilage Repair: A Systematic Literature Review. Joints 5:93-106

38. Sadr KN, Pulido P, McCauley JC, Bugbee W (2014) Fresh osteochondral allograft transplantation for osteochondritis dissecans of the knee. Orthopaedic Journal of Sports Medicine 2:

39. Sadr KN, Pulido PA, McCauley JC, Bugbee WD (2016) Osteochondral Allograft Transplantation in Patients With Osteochondritis Dissecans of the Knee. American Journal of Sports Medicine 44:2870-2875

40. Salai M, Ganel A, Horoszowski H (1997) Fresh osteochondral allografts at the knee joint: good functional results in a follow-up study of more than 15 years. Arch Orthop Trauma Surg 116:423-425

41. Schmidt KJ, Tirico LE, McCauley JC, Bugbee WD (2017) Fresh Osteochondral Allograft Transplantation: Is Graft Storage Time Associated With Clinical Outcomes and Graft Survivorship? American Journal of Sports Medicine 45:2260-2266

42. Shaha JS, Cook JB, Rowles DJ, Bottoni CR, Shaha SH, Tokish JM (2013) Return to an athletic lifestyle after osteochondral allograft transplantation of the knee. Am J Sports Med 41:2083-2089

43. Tirico LEP, McCauley JC, Pulido PA, Bugbee WD (2018) Lesion Size Does Not Predict Outcomes in Fresh Osteochondral Allograft Transplantation. Am J Sports Med;10.1177/0363546517746106363546517746106

44. Torga Spak R, Teitge RA (2006) Fresh osteochondral allografts for patellofemoral arthritis: long-term followup. Clin Orthop Relat Res 444:193-200

45. Wang D, Eliasberg CD, Wang T, Fader RR, Coxe FR, Pais MD, et al. (2017) Similar Outcomes After Osteochondral Allograft Transplantation in Anterior Cruciate Ligament-Intact and -Reconstructed Knees: A Comparative Matched-Group Analysis With Minimum 2-Year Follow-Up. Arthroscopy - Journal of Arthroscopic and Related Surgery 33:2198-2207

46. Wang D, Jones KJ, Eliasberg CD, Pais MD, Rodeo SA, Williams RJ, 3rd (2017) Condyle-Specific Matching Does Not Improve Midterm Clinical Outcomes of Osteochondral Allograft Transplantation in the Knee. Journal of Bone & Joint Surgery - American Volume 99:1614-1620

47. Wang D, Kalia V, Eliasberg CD, Wang T, Coxe FR, Pais MD, et al. (2018) Osteochondral Allograft Transplantation of the Knee in Patients Aged 40 Years and Older. Am J Sports Med 46:581-589

48. Wang D, Rebolledo BJ, Dare DM, Pais MD, Cohn MR, Jones KJ, et al. (2018) Osteochondral Allograft Transplantation of the Knee in Patients with an Elevated Body Mass Index. Cartilage;10.1177/19476035187546301947603518754630

49. Williams RJ, 3rd, Ranawat AS, Potter HG, Carter T, Warren RF (2007) Fresh stored allografts for the treatment of osteochondral defects of the knee. J Bone Joint Surg Am 89:718-726
